# Standard versus individualised positive end-expiratory pressure (PEEP) compared by electrical impedance tomography in neurocritical care: a pilot prospective single centre study

**DOI:** 10.1186/s40635-024-00654-3

**Published:** 2024-08-05

**Authors:** Vera Spatenkova, Mikulas Mlcek, Alan Mejstrik, Lukas Cisar, Eduard Kuriscak

**Affiliations:** 1 Neurocenter, Neurointensive Care Unit, Regional Hospital Liberec, Husova 357/10, 460 01 Liberec, Czech Republic; 2 Institute of Physiology, First Faculty of Medicine, Charles University in Prague, Albertov 5, 128 00 Prague, Czech Republic; 3 Department of Anaesthesia and Intensive Care, Third Faculty of Medicine, Charles University, Srobarova 50, 100 34 Prague, Czech Republic; 4 Faculty of Health Studies, Technical University of Liberec, Studentská 1402/2, 461 17 Liberec 1, Czech Republic; 5 2nd Department of Medicine-Department of Cardiovascular Medicine, Charles University in Prague, U nemocnice 2, 128 08 Prague, Czech Republic; 6 Technical Department, Regional Hospital Liberec, Husova 357/10, 460 01 Liberec, Czech Republic

**Keywords:** Positive end-expiratory pressure (PEEP), PEEP titration, Electrical impedance tomography, Ventilation homogeneity, Mechanical ventilation, Brain injury

## Abstract

**Background:**

Individualised bedside adjustment of mechanical ventilation is a standard strategy in acute coma neurocritical care patients. This involves customising positive end-expiratory pressure (PEEP), which could improve ventilation homogeneity and arterial oxygenation. This study aimed to determine whether PEEP titrated by electrical impedance tomography (EIT) results in different lung ventilation homogeneity when compared to standard PEEP of 5 cmH_2_O in mechanically ventilated patients with healthy lungs.

**Methods:**

In this prospective single-centre study, we evaluated 55 acute adult neurocritical care patients starting controlled ventilation with PEEPs close to 5 cmH_2_O. Next, the optimal PEEP was identified by EIT-guided decremental PEEP titration, probing PEEP levels between 9 and 2 cmH_2_O and finding the minimal amount of collapse and overdistension. EIT-derived parameters of ventilation homogeneity were evaluated before and after the PEEP titration and after the adjustment of PEEP to its optimal value. Non-EIT-based parameters, such as peripheral capillary Hb saturation (SpO_2_) and end-tidal pressure of CO_2_, were recorded hourly and analysed before PEEP titration and after PEEP adjustment.

**Results:**

The mean PEEP value before titration was 4.75 ± 0.94 cmH_2_O (ranging from 3 to max 8 cmH_2_O), 4.29 ± 1.24 cmH_2_O after titration and before PEEP adjustment, and 4.26 ± 1.5 cmH_2_O after PEEP adjustment. No statistically significant differences in ventilation homogeneity were observed due to the adjustment of PEEP found by PEEP titration. We also found non-significant changes in non-EIT-based parameters following the PEEP titration and subsequent PEEP adjustment, except for the mean arterial pressure, which dropped statistically significantly (with a mean difference of 3.2 mmHg, 95% CI 0.45 to 6.0 cmH_2_O, *p* < 0.001).

**Conclusion:**

Adjusting PEEP to values derived from PEEP titration guided by EIT does not provide any significant changes in ventilation homogeneity as assessed by EIT to ventilated patients with healthy lungs, provided the change in PEEP does not exceed three cmH_2_O. Thus, a reduction in PEEP determined through PEEP titration that is not greater than 3 cmH_2_O from an initial value of 5 cmH_2_O is unlikely to affect ventilation homogeneity significantly, which could benefit mechanically ventilated neurocritical care patients.

**Supplementary Information:**

The online version contains supplementary material available at 10.1186/s40635-024-00654-3.

## Background

Individualised bedside adjustment of mechanical ventilation is a very important strategy in acute coma neurocritical care patients. Since the brains of these patients are more susceptible to hypoxemia, hypo-, or hypercapnia, adjusting ventilatory parameters timely and individually could reduce secondary brain damage and improve outcomes [1–3]. Avoiding hypoxemia in the shortest possible time is essential since the injured brain of neurocritical patients, commonly affected by secondary damage, could quickly develop intracranial hypertension, which further worsens brain hypoxia [4–6].

It is well known that optimal positive end-expiratory pressure (PEEP) plays a key role in ventilator settings. It helps keep alveoli open, prevents alveolar derecruitment, contributes to optimal arterial oxygenation and brain tissue oxygen augmentation [7, 8], reduces intrapulmonary shunting [9], and improves pulmonary compliance by reducing lung ventilation inhomogeneities caused by atelectases and overdistensions. It is agreed that optimising PEEP could also be beneficial for patients with acute brain injury, comprising traumatic or non-traumatic conditions such as ischemic or haemorrhagic stroke and other acute brain tissue damage [10, 11].

There is still no fully settled evidence-based consensus regarding the optimal PEEP settings or PEEP titration strategy used during serious illness [12]. Patients with brain injury are ventilated to control proper oxygenation and to prevent secondary brain injury [1, 13–15]. They receive protective lung ventilation comprising a low tidal volume (VT), optimised levels of PEEP, and possibly recruitment manoeuvres to prevent lung or brain damage, as inadequate mechanical ventilation could inflict further cerebral and pulmonary damage [2, 5, 16, 17]. These patients are, however, not commonly included in studies focusing on mechanical ventilation [18, 19], and protective ventilation that benefits patients unaffected by acute brain tissue damage could have harmful effects on those affected. For instance, inappropriate controlled ventilation with too low tidal volumes could cause hypercapnia. The high partial pressure of CO_2_ in arterial blood (PaCO_2_) is a potent cause of cerebrovascular reactivity, and unwanted vasodilation of cerebral arteries could increase intracranial pressure [3]). On the other hand, hyperventilation causes hypocapnia, resulting in vasoconstriction, unwanted decrease in cerebral blood flow and possible brain hypoxia [3, 20]. Moreover, high PEEP values or inappropriate recruitment manoeuvres could increase intrathoracic pressure, reduce venous return, decrease the mean arterial pressure (MAP) and cerebral venous outflow, elevating herewith the intracranial pressure [21–23] and compromising cerebral perfusion pressure [5, 13, 24–28]. A recommended PEEP for neurocritical care patients starts at 5 cmH_2_O but can be as high as 10 or even 15 cmH_2_O if there is no intracranial pressure increase [6, 11].

A commonly used approach to find an optimal PEEP is to look for the pressure of the highest lung compliance [29]. Also, a pressure–volume curve [30], global inhomogeneity index (GIi) [31], dead space (lowest dead space to tidal volume fraction [32]), or other lung-related parameters such as arterial oxygenation [33], the difference between partial pressure of arterial O_2_ and end-tidal partial pressure of CO_2_ (EtCO_2_) [34, 35], stress index [36, 37], oesophageal manometry [38, 39] or ultrasound [40], can be used to titrate the PEEP.

One of the widely used approaches to set up the PEEP is to balance alveolar collapse against overdistension [38, 41, 42], assessed by electrical impedance tomography (EIT, radiation-free, non-invasive, bedside, and continuous lung imaging [43, 44]), being close to titration of best compliance [29, 42]. Finding a balance between alveolar collapse and overdistension could be a good compromise, resulting in less lung injury compared to approaches that minimise overdistensions [45].

This study investigates whether individualised PEEP levels, titrated by the EIT and differing by less than 3 cmH_2_O from the standard PEEP set to 5 cmH_2_O, result in significant changes in ventilation homogeneity in acute neurocritical patients with healthy lungs, as assessed using integrated EIT software. The main question we addressed was how necessary it is to titrate the PEEP by the EIT when the initial value of PEEP is set close to 5 cmH_2_O. We did not systematically assess which PEEP values probed during the PEEP titration were most optimal regarding the EIT-derived parameters of ventilation homogeneity. To our knowledge, this study presents the first investigation focusing on the effect of titrated PEEPs smaller than 5 cmH_2_O, evaluated in neurointensive care unit (NICU) patients with healthy lungs, on EIT-derived parameters showing ventilation homogeneity.

## Methods

This prospective single-centre study was conducted at the 18-bed NICU of the Neurocenter of the Regional Hospital with 900 beds. The study was performed in part C of the NICU with six separate beds in boxes for acute neurological and neurosurgical intensive care patients with controlled ventilation using a Hamilton G5 ventilator.

During the years 2019–2021, we included 55 acute coma neurocritical care patients (demographic and clinical data are shown in Table 1) who fulfilled the criteria: 1/ adult ≥ 18 years old; 2/ mechanical ventilation on admission; 3/ acute brain disease; 4/ free EIT device and size of belts; 5/ hemodynamic stability; 6/ without intracranial hypertension. This study had the following exclusion criteria: 1/ age < 18 years; 2/ intracranial hypertension; 3/ hemodynamic instability; 4/ terminal stage of the disease.

**Table 1:** Demographic and clinical data of our study population

Parameter (*N* = 55), unit	% (*N*) Mean (SD) Median (IRQ)
Age, years	68 (56–74)
Sex, male, pts	60.0% (33)
Weight, kg	85 (74–95)
Body Mass Index, unitless	26.9 (24.8–32.9)
Ideal Body Weight, kg	65.2 (57.4–71.1)
Admission	
Primary, pts	18.2% (10)
Secondary to 24 h, pts	47.3% (26)
Secondary after 24 h, pts	34.5% (19)
Acute admission, %, pts	76.4% (42)
Stay in the NICU, days	12 (9–16)
Diagnoses	
Stroke, pts	56.4% (31)
Trauma, pts	20.0% (11)
Tumour, pts	9.1% (5)
Epilepsy, pts	12.7% (7)
Infection, pts	1.8% (1)
Operation, pts	67.3% (37)
Scores, unitless	
TISS on admission	33 (31–37)
APACHE II on admission	19 (16–23)
GCS onset brain disease	13 (8–15)
GCS on admission	6 (3–8)
GOS on NICU discharge	2 (2–2)
Analgosedation	
Sufentanil, pts	100.0% (55)
Propofol, pts	70.9% (39)
Midazolam, pts	85.5 (47)
Dexmetomidine, pts	12.7% (7)
Neuromonitoring	
Intracranial pressure, pts	10.9% (6)
Cerebral tissue oximetry, pts	7.3 (4)
Cerebral microdialysis, pts	5.5% (3)
Bispectral index, pts	40.0% (22)

*APACHE* Acute Physiology and Chronic Health Evaluation, *GCS* Glasgow Coma Scale, *GOS* Glasgow Outcome Scale, *IQR* InterQuartile Range, *NICU* Neurointensive Care Unit, *pts* number of patients, *SD* Standard Deviation, *TISS* Therapeutic Intervention Scoring System

All patients were mechanically ventilated, mostly with Adaptive Support Ventilation (ASV, 39 patients) and Duo Positive Airway Pressure (DuoPAP, 16 patients), with an initial value of PEEP set to 5 cmH_2_O upon admission to NICU. Target values of ventilation parameters were as follows: minute ventilation set at 90–130% of ideal body weight, adjusted continuously based on pCO_2_ levels, peripheral capillary Hb saturation (SpO_2_) maintained above 94% with a fraction of inspired oxygen (FiO_2_) between 30 and 40% (and up to 100% when it is necessary to maintain the SpO_2_). The target respiratory rate (RR) in DuoPAP mode was 18–20 breaths per minute (bpm), and the value of high positive airway pressure was 22 cmH_2_O. The ventilator in DuoPAP mode adjusted the VT automatically to reach the mentioned targets, while in the ASV mode, the breathing pattern was controlled by adjusting the inspiratory pressure, VT and RR, to achieve the targets by optimising the work of breathing. Both ASV and DuoPAP modes supported spontaneous breaths, with a flow trigger set to 1–3 L/min. All patients underwent the protocol described below, during which the ventilator settings remained unchanged, including the periods of readout times used to evaluate the EIT-based parameters, except for the PEEP value, which changed during the PEEP titration procedure and at the subsequent adjustment of PEEP.

**Fig. 1:**
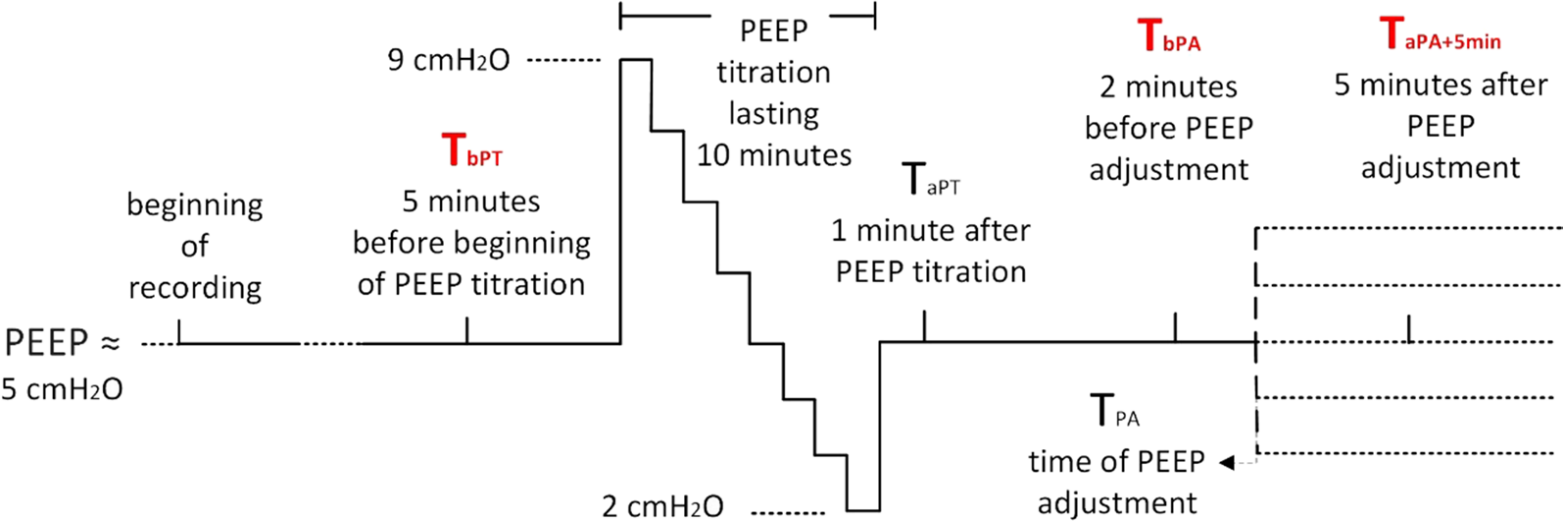
PEEP titration and examination protocol. This figure shows the examination protocol with PEEP starting at around 5 cmH_2_O, followed by the PEEP titration and subsequent change (adjustment) of PEEP to the optimal value found by PEEP titration. At each time instance, the readout times T_bPT_, T_bPA_, T_aPA+5min_, the EIT-derived parameters were averaged over 1-min intervals. *EIT *Electrical Impedance Tomography, *PEEP* Positive End-Expiratory Pressure, *T*_*bPT*_ readout time 5 min before PEEP titration procedure, *T*_*bPA*_ readout time 2 min before PEEP adjustment, *T*_*aPA*+*5min*_ readout time 5 min after PEEP adjustment

1. The PEEP titration procedure was carried out using the EIT device (Timpel EIT Enlight 1800) approximately 2 days after placing an EIT belt on the patient’s chest (beginning of continuous EIT measurement). The decremental PEEP titration procedure started by changing the actual values of PEEP that were close 5 to cmH_2_O (max 8 cmH_2_O, min 3 cmH_2_O, mean 4.75 cmH_2_O ± standard deviation (SD) 0.94, measured 5 min before the PEEP titration, see Table 2 for details), to 9 cmH_2_O, which decreased by one cmH_2_O decrements to 2 cmH_2_O, after which the PEEP was set again to values found before the PEEP titration. The SD of ΔPEEP before and after titration, measured by a pressure sensor attached to the EIT machine, was 0.43 cmH_2_O and reflects the accuracy of the sensor, the particular arrangement of ventilator tubing circuit and the ability of the ventilator to maintain preset PEEP values. On average, each PEEP step lasted 50 s, and the entire titration lasted 10 min, see Fig. 1 and Table 2 for details on PEEP titration.
2. During the PEEP titration, an optimal PEEP was determined using a Timpel PEEP titration tool to minimize overdistension and collapse evaluated by the EIT [42]. It should be mentioned that such crossing-point PEEPs obtained during a decremental PEEP titration under the condition of muscle paralysis can have different effects on the lungs during spontaneous breathing, which is discussed in the Discussion section.
3. The identified optimal PEEP was then set on the ventilator and maintained for at least one hour.

**Table 2:** PEEP titration parameters, ventilation parameters and ventilation regimes

Parameter, unit	% (*N*) Mean (SD) Median (IRQ)			
Patients with PEEP titration, pts	100% (55)			
Patients with PEEP adjusted (changed) after PEEP titration, pts	85% (47)			
Patients whose PEEP has not changed after PEEP titration^†^, pts	14% (8)			
Duration of PEEP titration procedure, minutes	10 (2)			
Duration of each PEEP step during PEEP titration, seconds	50 (0)			
PEEP at the beginning of controlled ventilation, cmH_2_O	0.95 (0.54)			
PEEP 5 min before PEEP titration (T_bPT_), cmH_2_O	4.75 (0.94)			
3 cm H_2_O, pts	1.8% (1)			
4 cm H_2_O, pts	9% (5)			
5 cm H_2_O, pts	78% (43)			
6 cm H_2_O, pts	5.4% (3)			
7 cm H_2_O, pts	3.6% (2)			
8 cm H_2_O, pts	1.8% (1)			
PEEP 5 min after PEEP adjustment (T_aPA+5 min_), cmH_2_O	4.26 (1.5)			
2 cm H_2_O, pts	7.2% (4)			
3 cm H_2_O, pts	27% (15)			
4 cm H_2_O, pts	20% (11)			
5 cm H_2_O, pts	20% (11)			
6 cm H_2_O, pts	16% (9)			
7 cm H_2_O, pts	3.6% (2)			
8 cm H_2_O, pts	5.4% (3)			
Pair-wise changes in parameters between readout events T_bPT_, T_bPA_, and T_aPA+5 min_	T_bPT_: T_aPA+5 min_	T_bPT_: T_bPA_		T_bPA_: T_aPA+5 min_
ΔVT, mL	+ 21 (53)	+ 15 (51)		+ 6 (43)
ΔPEEP, cmH_2_O	−0.48 (1.6)	−0.45 (0.43)		−0.03 (1.96)
Δ(PIP-PEEP), cmH_2_O	+ 0.7 (1.7)	+ 0.6 (1.6)		+ 0.1 (1.3)
ΔRR, bpm	−0.32 (2.1)	−0.27 (2.1)		−0.05 (1.1)
Values of VT | PEEP | PIP-PEEP | RR, mL | cmH_2_O | cmH_2_O | bpm	VT	PEEP	PIP-PEEP	RR
5 min before PEEP titration (T_bPT_)	520 (102)	4.75 (0.94)	14 (2.9)	16 (3.0)
2 min before PEEP adjustment (T_bPA_)é	535 (102)	4.29 (1.24)	15 (3.2)	16 (2.5)
5 min after PEEP adjustment (T_aPA+5 min_)	541 (107)	4.26 (1.5)	13 (3.0)	16 (2.5)
ASV regime^‡^, pts	71% (39)			
Values of VT | PIP-PEEP | RR, mL | cmH_2_O | bpm	VT	PIP-PEEP		RR
5 min before PEEP titration	510 (104)	13.4 (3.1)		16.2 (3.1)
2 min before PEEP adjustment	516 (94)	13.9 (3.2)		15.7 (2.1)
5 min after PEEP adjustment	517 (98)	13.8 (3.0)		15.7 (2.2)
DuoPAP regime^‡^, pts	29% (16)			
Values of VT | PIP-PEEP | RR, mL | cmH_2_O | bpm	VT	PIP-PEEP		RR
5 min before PEEP titration	543 (93)	15.0 (2.1)		16.1 (3.0)
2 min before PEEP adjustment	580 (108)	16.0 (2.8)		16.3 (3.2)
5 min after PEEP adjustment	597 (109)	16.4 (2.2)		16.3 (3.1)
Time between the end of PEEP titration and setting the optimized PEEP, minutes	18 (3–32)			
Time between NICU admission and enrolment (beginning) of EIT recording, days	2 (1—3)			
Duration of EIT recording, days	3 (3—5)			
Patients being intubated upon admission to the NICU, pts	58% (32)			
Patients with spontaneous ventilation before intubation in NICU, pts	42% (23)			
Duration of spontaneous ventilation before intubation, hours	16 (4—40)			
Parameters at the beginning of mechanical ventilation				
pCO_2_^†^, kPa	4.8 (0.6)			
FiO_2_, %	41 (8)			
SpO_2_, %	98.2 (1.6)			
EtCO_2_, kPa	4.4 (0.59)			
C_stat_, mL/cmH_2_O	53 (20)			
Averaged parameters over the course of mechanical ventilation				
pCO_2_, kPa	4.98 (0.85)			
FiO_2_, %	36 (0.09)			
SpO_2_, %	97.1 (0.82)			
EtCO_2_, kPa	4.7 (0.54)			
C_stat_, mL/cmH_2_O	53 (13)			

*ASV* Adaptive Support Ventilation, *DuoPAP* Duo Positive Airway Pressure, *bpm* breaths per minute, *C*_*stat*_ static lung mechanical compliance from the ventilator, *EtCO*_*2*_ End-tidal partial pressure of CO_2_, *FiO*_*2*_ a fraction of inspired oxygen, *IQR* InterQuartile Range, *NICU* Neuro-Intensive Care Unit, *pCO*_*2*_ partial pressure of carbon dioxide in the blood, *PEEP* Positive End-Expiratory Pressure, *RR* Respiratory Rate, *SD* Standard Deviation, *SpO*_*2*_ peripheral capillary Hb-Saturation with oxygen measured by pulse oximetry, *T*_*bPT*_ readout time 5 min before the PEEP titration, *T*_*bPA*_ readout time 2 min before the PEEP adjustment, *T*_*aPA*+*5min*_ readout time 5 min after the PEEP adjustment

^†^pCO_2_ was not necessarily measured at the same hour as was the beginning of mechanical ventilation in the NICU but approximately every 8 h. The first measured value was used to calculate the mean (SD)

^‡^Ventilation settings stayed unchanged during the PEEP titration period and subsequent PEEP change in evaluated observations

We examined how the EIT-derived parameters, as well as other selected parameters described below, changed between the time instances (readout times or readout periods) described below:

1. T_bPT_—begins 5 min before the beginning of PEEP titration
2. T_bPA_—begins 2 min before the PEEP adjustment, or more precisely, 2 min before the change of PEEP to the optimal value obtained by the PEEP titration procedure
3. T_aPA+5min_—begins 5 min after the PEEP has been changed/adjusted to optimal values found by the PEEP titration. This 5-min duration sufficed to observe the effects resulting from adjusting the PEEP levelling off [46]. The described readout times used in our protocol and other details of the PEEP titration procedure are depicted in Fig. 1. Each of the abovementioned readout periods lasted one minute, during which the registered parameters (most of them evaluated for every breath) were averaged.

Sporadic spontaneous breaths were identified through detailed visual inspection of continuous pressure and flow/volume curves recorded by EIT sensors during the abovementioned one-minute readout intervals in seven patients. Therefore, the readout times of evaluated data segments were shifted by up to 4 min to find periods without spontaneous breaths. We detected spontaneous breaths 5 min before the beginning of PEEP titration (at T_bPT_, found in seven patients), none of these were observed 2 min before the adjustment of PEEP (at T_bPA_), and some were observed 5 min after the adjustment of PEEP (at T_aPA+5min_, found in two patients).

During the controlled ventilation, all patients were in analgosedation (see Table 1 for details) and received Atracurium for myorelaxation right before the PEEP titration. Among 55 patients, eight had their initial PEEP assessed as optimal and was not adjusted, while in 47 patients, the PEEP was adjusted to the optimal value found by the PEEP titration procedure (see Table 2 for details).

### EIT analysis

All patients were equipped with an EIT belt fit according to their chest size, utilizing a range of six different belt sizes. Each belt contained 16 evenly spaced electrodes, where one pair of electrodes applied an alternating current while the others measured it. The pattern of applied and recording electrodes automatically cycled to get an apparent rotatory movement of the electrodes to create a tomography-like imaging, providing a real-time reconstruction of regional, pixel-wise lung impedance represented by a 32 × 32 matrix updated every 20 ms [43]. All EIT frames were filtered with a 0.03 Hz to 0.7 Hz passband filter to eliminate impedance changes caused by the heart's electrical activity. All EIT frames and other EIT-based parameters were processed using MATLAB R2019b software. Most EIT-based parameters were derived from spatiotemporal, pixel-wise characteristics of lung tissue impedance evaluated throughout every breath. This enabled detailed analysis and mutual comparison of various parameters between consecutive breaths and various readout intervals.

The following EIT-derived parameters, assessing the inhomogeneity of regional lung ventilation, were continuously evaluated for every single breath:

- GIi (Global Inhomogeneity index, [32, 47]), examines the spatial heterogeneity of distribution of tidal volume inside the lungs and was calculated as:

Σ |ΔZ_pix_ – median(ΔZ_all_pixels_)| / Σ (ΔZ_pix_), where ΔZ_pix_ represents the change in electrical impedance Z during the breathing cycle in a specific lung region corresponding to one pixel of the EIT matrix (i.e., the tidal change in electrical impedance of the respective lung pixel). Here, Σ denotes the sum over all lung pixels. GIi was calculated for the entire lungs, as well as separately for the left and right lungs. The higher the value of GIi, the greater the ventilation inhomogeneity in the evaluated lung region.

- Regional ventilation delay inhomogeneity (RVDI, [44, 48]), which assesses the temporal heterogeneity of inflation inside the lungs during each breathing cycle, was calculated as:

RVDI = SD (RVDi of all pixels),

where RVDi represents the RVD index of each pixel, defined as (Δt_pix_^40%^ /(t_max_-t_min_)) x 100%. Here, Δt_pix_^40%^ is the time from the inspiration to reach 40% of the pixel’s maximal impedance, and t_max_−t_min_ is the pixel’s inflation time. A higher RVDI indicates less homogenous temporal air distribution within the specified region of interest (in our study, encompassing the entire lungs).

- Dynamic compliance was calculated from pressure and flow sensors integrated into the EIT device as C_dyn_ = VT/(PIP ‑ PEEP), where PIP is the Peak Inspiratory Pressure.
- Electrical compliance—C_el_, calculated as ‘ΔZ of both lungs’/(PIP ‑ PEEP)—which can be considered an electrical surrogate of the regional lung compliance as described in detail in [42].
- Four Quadrants ventilation Inhomogeneity index (4QIi)—we introduced this measure of ventilation homogeneity, calculated for each breath as the standard deviation of ΔZ for four quadrants [49–51] comprising the entire lungs. Each quadrant ΔZ is measured as a percentage of the total ΔZ of all quadrants (100%), where the quadrants correspond to the ‘right anterior’, ‘right posterior’, ‘left anterior’, and ‘left posterior’ portions of the horizontal chest section. The higher the value of 4QIi, the less evenly distributed the ventilation to these four quadrants.
- Lung volume and pressure curves were obtained from Timpel flow and pressure sensors (inserted in a ventilator tubing circuit with a sampling rate of 50 Hz), from which the VT, PEEP, and PIP values were calculated for each breath.

We evaluated how much the abovementioned EIT-derived parameters changed due to the PEEP titration procedure, due to the setting of the new optimal PEEP found by the titration, and due to the combined effect of both these events. This was quantified by analysing changes between the three readout times T_bPT_, T_bPA_, and T_aPA+5min_, as explained in the previous paragraphs.

In contrast to the abovementioned EIT-based parameters monitored on a breath-by-breath basis, the following parameters were monitored by our nurses and recorded hourly in each patient’s medical record: SpO_2,_ EtCO_2_, central venous pressure (CVP), MAP, stroke volume variation (SVV), cardiac index (CI, obtained by Edwards Vigileo or EV1000), inspiratory resistance, and static compliance (C_stat_), the last two obtained from the ventilator. The update rate of these eight parameters and the update rate of EIT-derived parameters described in the previous paragraph were quite disproportionate, so the respective parameters measured at corresponding readout times could not be compared with a better time resolution than one hour.

### Statistical analysis

The basic descriptive statistics was done in Microsoft Excel. Inferential analysis was evaluated in Matlab R2019b. Continuous parameters are reported as mean ± SD, counts and percentages, median and IQR (interquartile range). The normality of the distribution of the analysed variables was assessed using the Shapiro–Wilk test. Depending on the normality of the data, paired samples were compared using either the paired t-test or the Wilcoxon test. For comparisons involving more than two samples, we employed a one-way repeated measures ANOVA test. Post-hoc pairwise comparisons were analysed using Tukey’s test. All tests were considered statistically significant at the 0.05 significance level.

## Results

We examined the effect of PEEP titration on the ventilation homogeneity measured by EIT in 55 acute coma neurocritical care patients with healthy lungs. The median age of our cohort was 68 years (IQR 56–74), with 60% of males. The median time spent in the NICU was 12 days (IQR 9–16), and the median EIT recording time was 3 days (IQR 3–5 days), see Tables 1 and 2 for further details. During the mechanical ventilation, the mean SpO_2_ was 97.1 ± 0.82%, and the mean pCO_2_ was 4.98 ± 0.85 kPa. In the ASV regime, the mean PIP and RR were 18.1 cmH_2_O and 15.9 bpm, respectively, and in the DuoPAP regime, the mean PIP was 20.4 cmH_2_O and the mean RR was 16.2 bpm. At the beginning of controlled ventilation, the mean pCO_2_ was 4.8 ± 0.6 kPa, SpO_2_ was 98.2 ± 1.6%, FiO_2_ was 41 ± 8%, PEEP was 4.95. ± 0.54 cmH_2_O and static compliance was 53 ± 20 cmH_2_O (see Tables 2 and 3). Five minutes before the PEEP titration procedure, the mean PEEP value was 4.75 ± 0.94 cmH_2_O. Afterwards, the PEEP titration procedure started to determine the new optimal PEEP, which was then set on the ventilator minutes after the end of PEEP titration (median 18 min, IQR 3–32 min), ranging from 2 to 8 cmH_2_O (4.26 ± 1.5 cmH_2_O, see Table 2 for further details). The maximum absolute change in PEEP (|∆PEEP|) at PEEP adjustment was three cmH_2_O, the mean value of |∆PEEP| 1.7 cmH_2_O and SD of ∆PEEP = 1.96 cmH_2_O.

**Table 3:** The effect of PEEP titration and subsequent adjustment of PEEP on measured parameters

Parameter, unit	T_bPT_ mean (SD)	T_bPA_ mean (SD)	T_aPA+5 min_ mean (SD)	T_bPT_ vs. T_aPA+5 min_^†^	T_bPT_ vs. T_bPA_^‡^	T_bPA_ vs. T_aPA+5 min_^§^
	All patients with PEEP titration (55 patients)					
	Values at three different readout times:			*p*-values of Tukey’s multiple comparison test:		
GIi both lungs, unitless	0.84 (0.08)	0.85 (0.078)	0.83 (0.077)	0.056	0.0065**	0.34
GIi right lung, unitless	0.83 (0.077)	0.84 (0.077)	0.84 (0.077)	0.17	0.042*	0.13
GIi right lung, unitless	0.84 (0.094)	0.84 (0.11)	0.84 (0.11)	0.44	0.073	0.70
RVDI both lungs, %	9.5 (6.5)	8.5 (7)	0.84 (0.11)	0.56	0.32	0.71
4QIi, %	5.6 (2.9)	6.2 (2.9)	6.3 (2.8)	0.003**	0.0036**	0.54
C_dyn_, mL/cmH_2_O	39 (12)	39 (13)	39 (14)	0.99	0.87	0.33
PEEP, cmH_2_O	4.75 (0.94)	4.3 (1.2)	4.26 (1.5)	0.074	0.065	0.99
PIP-PEEP, cmH_2_O	14 (2.9)	15 (3.2)	15 (3.0)	0.0085**	0.0064**	0.97
VT, mL	520 (102)	535 (102)	541 (107)	0.013*	0.082	0.56
SD of ΔPEEP				1.6 cmH_2_O	0.43 cmH_2_O	1.96 cmH_2_O
SD of ΔVT				53 mL	51 mL	43 mL
	A subgroup of patients with PEEP titration and subsequent adjustment in PEEP (47 patients)					
	Values at three different time instances:			*p*-values of Tukey’s multiple comparison test:		
GIi both lungs, unitless	0.84 (0.08)	0.84 (0.08)	0.84 (0.08)	0.13	0.028*	0.55
GIi right lung, unitless	0.83 (0.08)	0.84 (0.08)	0.84 (0.08)	0.21	0.09	0.27
GIi right lung, unitless	0.83 (0.08)	0.84 (0.11)	0.84 (0.11)	0.71	0.23	0.76
RVDI both lungs, %	8.8 (5.8)	8.1 (6.8)	8.4 (6.7)	0.89	0.66	0.72
4QIi, %	5.5 (2.9)	6.0 (2.9)	6.2 (2.9)	0.01*	0.02*	0.47
C_dyn_, mL/cmH_2_O	39 (12)	39 (13)	39 (14)	0.99	0.71	0.27
PEEP, cmH_2_O	4.8 (1)	4.5 (1.2)	4.2 (1.6)	0.069	0.08	0.59
PIP-PEEP, cmH_2_O	14 (2.9)	14 (3.0)	15 (3.0)	0.01*	0.054	0.25
VT, mL	520 (101)	526 (91)	541 (106)	0.035*	0.62	0.019*

	T_bPT_ mean (SD)	T_aPT_^a^ mean (SD)	T_aPT+5min_^a^ mean (SD)	T_bPT_ vs. T_aPT+5min_^a^	T_bPT_ vs. T_aPT_^a^	T_aPT_ vs. T_aPT+5min_^a^
	A subgroup of patients with PEEP titration without subsequent adjustment in PEEP (8 patients^a^)					
	Values at 3 different time instances:			*p*-values of Tukey’s multiple comparison test:		
GIi both lungs, unitless	0.85 (0.04)	0.86 (0.04)	0.86 (0.04)	0.21	0.21	0.51
GIi right lung, unitless	0.85 (0.04)	0.86 (0.04)	0.86 (0.04)	0.76	0.48	0.56
GIi right lung, unitless	0.85 (0.05)	0.87 (0.05)	0.87 (0.05)	0.13	0.15	0.90
RVDI both lungs, %	14.0 (8.5)	11.0 (8.0)	11.0 (8.2)	0.17	0.15	0.45
4QIi, %	6.4 (3.1)	6.9 (2.8)	7.0 (2.7)	0.12	0.11	0.23
C_dyn_, mL/cmH_2_O	37 (10)	38 (10)	38 (10)	0.11	0.07	0.88
PEEP, cmH_2_O	4.5 (0.2)	4.6 (0.2)	4.6 (0.2)	0.63	0.67	0.99
PIP-PEEP, cmH_2_O	15 (3.3)	15 (3.5)	15 (3.5)	0.78	0.84	0.89
VT, mL	523 (114)	544 (111)	542 (112)	0.039*	0.019*	0.72

*C*_*dyn*_ dynamic lung mechanical compliance, *GIi* Global Inhomogeneity index, *PEEP* Positive End-Expiratory Pressure, *PIP* Peak Inspiratory Pressure, *RVDI* Regional Ventilation Delay Inhomogeneity, *SD* Standard Deviation, *VT* Tidal Volume, *4QIi* Four Quadrants Ventilation Inhomogeneity index, ΔPEEP, *ΔVT* difference between respective values at two given readout times, *T*_*bPT*_ readout time 5 min before the PEEP titration, *T*_*bPA*_ readout time 2 min before the PEEP adjustment, *T*_*aPA*+*5min*_ readout time 5 min after the PEEP adjustment

^a^in this group of patients, the PEEP remained unchanged after the PEEP titration: the T_aPT_ corresponds to the readout time one minute after the PEEP titration and T_aPT+5min_ to the readout time 5 min after the PEEP titration

^*^p < 0.05, **p < 0.01, ***p < 0.001

^†^Comparison between parameters 5 min before PEEP titration and 5 min after PEEP adjustment

^‡^Comparison between parameters 5 min before PEEP titration and 2 min before PEEP adjustment

^§^Comparison between parameters 2 min before PEEP adjustment and 5 min after PEEP adjustment

**Fig. 2:**
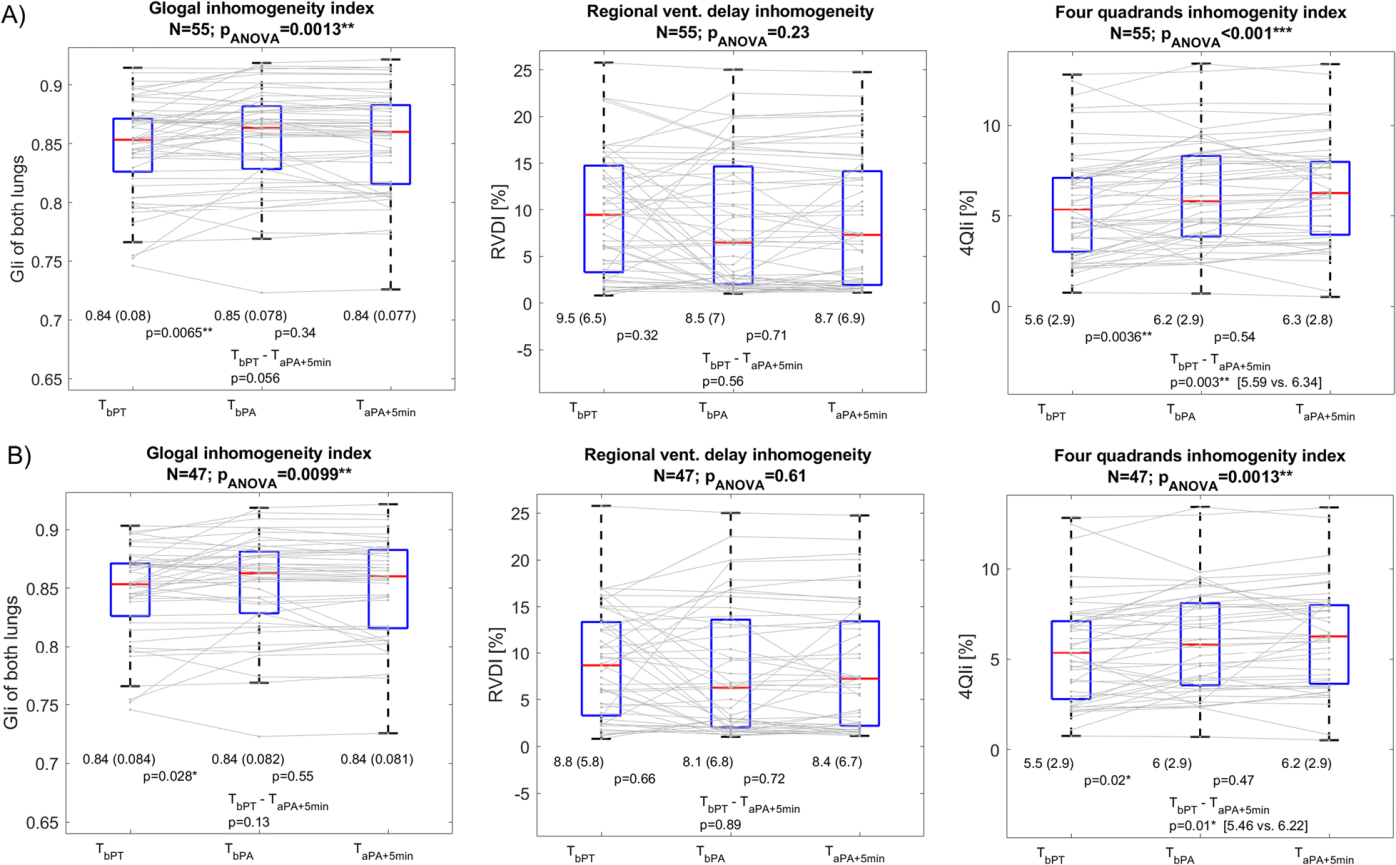
EIT-derived parameters of ventilation homogeneity. Boxplots show how much the EIT-derived parameters of ventilation homogeneity changed between measurements taken 5 min before the PEEP titration (T_bPT_), 2 min before the PEEP adjustment (T_bPA_), and 5min after the PEEP adjustment (T_aPA+5min_). Panel **A** shows the parameters for all 55 enrolled patients. Panel **B** shows the parameters for a subgroup of 47 patients whose PEEP values changed after the PEEP titration at the time of subsequent PEEP adjustment. Mean values (standard deviations) of measured parameters, the *p*-values of one-way repeated measures ANOVA (in the caption) and the *p*-values of post-hoc Tukey multi-comparison tests (inside boxplots), comparing pairs of respective parameters between given readout times can be seen, along with the medians (red bars), quartiles Q1- Q3 (blue boxes), and 99.3 percentile ranges indicated by whiskers. *p < 0.05, **p < 0.01, ***p < 0.001. *GIi* Global Inhomogeneity index, *RVDI* Regional Ventilation Delay Inhomogeneity, *4QIi* Four Quadrants ventilation Inhomogeneity index

In Fig. 2 and Table 3, the EIT parameters of ventilation homogeneity—the GIi and the RVDI of the entire lungs and the 4QIi are shown evaluated 5 min before the PEEP titration (T_bPT_), 2 min before the PEEP adjustment (T_bPA_), and 5 min after the PEEP adjustment (T_aPA+5min_). Here, we can see how they changed between respective readout times in all 55 enrolled patients (Panel A) undergoing the PEEP titration procedure. The only parameter of ventilation homogeneity that changed significantly due to the combined effects of PEEP titration and PEEP adjustment (between T_bPT_ and T_aPA+5min_) was the 4QIi, which increased from 5.6% ± 2.9% to 6.3% ± 2.8%, with a *p*-value of 0.003, calculated by Tukey post-hoc test. The GIi evaluated separately for the right and left lung, and the electrical compliance of the entire lungs is shown in Supplement, Fig. S1, Panel A.

Analysing the subgroup of 47 patients whose PEEP was adjusted after the PEEP titration resulted in a similar finding. Only the 4QIi changed significantly between the T_bPT_ and T_aPA+5min_, increasing from 5.5% ± 2.9% to 6.2% ± 2.9% (*p* = 0.01, see Fig. 2B and Table 3 for more details). Figure 2 also focuses on the isolated effects of PEEP titration and PEEP adjustment on EIT-derived parameters. It shows that the effect of PEEP adjustment alone on the measured parameters of ventilation homogeneity was statistically insignificant in all 55 patients and also in the subgroup of 47 patients whose PEEPs changed after the PEEP titration (*p*-values of all parameters were bigger than 0.05 for both groups, see Table 3). This indicates that the statistically significant change in 4QIi caused by both interventions—the PEEP titration and subsequent PEEP adjustment—was primarily driven by the PEEP titration alone. Significant *p*-values between the T_bPT_ (5 min before the PEEP titration) and T_bPA_ (2 min before the PEEP adjustment) demonstrate this observation regarding the 4QIi (*p* = 0.0036) and GIi (*p* = 0.0065) parameters in the entire cohort of 55 patients, as well as in the subgroup of 47 patients (*p* = 0.02 and *p* = 0.028, respectively). Analysing a subgroup of 8 patients exposed only to the PEEP titration procedure, without the subsequent adjustment of PEEP, did not show any statistically significant effect on measured parameters between the pairs of readout times before and after the titration (see Table 3 and Fig. S2).

Considering that 39 patients were under the ASV and 16 under the DuoPAP ventilation regime during our study protocol, we separately assessed all respective EIT-derived parameters for these groups. For both ASV and DuoPAP regimes, the GIi and 4QIi exhibited statistically significant changes between the T_bPT_ and Ta_PA+5min_ (see Fig. S3, S4 and Table S1). Again, statistically significant changes were mainly caused by the PEEP titration procedure, whereas the subsequent PEEP adjustment showed only statistically insignificant effects.

Considering that the tidal volume varied slightly due to an automatic adjustment of the ventilator between the T_bPT_ and T_aPA+5min_ (for instance, across all 55 patients, the mean tidal volume increased by 21 mL, from 520 to 541 mL, see Tables 2 and 3 for details), we analysed 28 patients whose tidal volume changed by less than 20 mL during this interval. Here, only the 4QIi showed a statistically significant change between the T_bPT_ and T_aPA+5min_ (increasing from 6.19% ± 3.0% to 7.14% ± 2.9%, *p* = 0.0084, as shown in Fig. S5), while again, none of the measured EIT-based parameters changed significantly due to the PEEP adjustment between the T_bPA_ and T_aPA+5min_ (shown in supplementary Table S1). In a subset of 27 patients where VT increased by more than 20 mL between the T_bPT_ and T_aPA+5min_, both GIi and RVDI showed statistically significant changes due to the effect of PEEP titration alone (*p* < 0.001 for GIi and *p* = 0.048 for RVDI). The subsequent PEEP adjustment did not significantly alter the EIT-based measured parameters (see Fig. S6 and Table S1).

Next, since the overall change in PEEP brought by the PEEP adjustment was quite small (the maximum change in PEEP at PEEP adjustment was three cmH_2_O as mentioned), we selected 14 patients whose PEEP changed by two or more cmH_2_O and 41 patients whose PEEP changed by less than 2 cmH_2_O (|ΔPEEP|≥ 2 cmH_2_O) at PEEP adjustment between the T_bPT_ and T_aPA+5min_. None of the respective EIT-based parameters showed statistically significant changes due to the effect of PEEP titration itself (between T_bPT_ and T_bPA_), due to the subsequent PEEP adjustment (between T_bPA_ and T_aPA+5min_), or when both events were considered together (between T_bPT_ and T_aPA+5min_, see Fig. S7 and Table S1).

Regarding the parameters manually registered by nurses every hour, we did not observe any statistically significant changes in SpO_2_, EtCO_2_, CVP, C_stat_, IR, SVV and CI due to the PEEP titration and subsequent PEEP adjustment (see Fig. S8 and Table 4). An interesting finding is a statistically significant decrease in MAP by 3.2 mm Hg (from 86.0 ± 8 to 82.8 ± 10 mmHg, 95% CI 0.45–6.0 cmH_2_O, *p* < 0.001) in our cohort of 55 patients.

**Table 4:** Non-EIT-based parameters obtained with hourly resolution

Parameter, unit	Before PEEP titration^†^ Mean (SD)	After PEEP adjustment^†^ Mean (SD)	*p*-value (paired t-test) (see Fig. S8 and Methods for more information)
SpO_2_, %	98.0 (1.8)	97.8 (2.0)	0.41
EtCO_2_, kPa	4.74 (0.63)	4.75 (0.62)	0.89
CVP, cmH_2_O	10.8 (3.0)	10.5 (4.2)	0.49
MAP, mmHg	86.0 (8.1)	82.8 (10.0)	< 0.001
SVV, %	8.02 (4.3)	4.02 (4.0)	0.99
CI, L/min/m^2^	3.37 (1.5)	2.19 (1.0)	0.25
IR^‡^, cmH_2_O/L/s	17.8 (4.8)	17.1 (5.2)	0.23
C_stat_^‡^, mL/cmH_2_O	49.4 (19)	52.9 (25)	0.062

*CI* Cardiac Index, *CVP* Central Venous Pressure, *C*_*stat*_ static lung mechanical compliance, *EtCO*_*2*_ End-tidal partial pressure of CO_2_, *IR* Inspiratory Resistance, *MAP* Mean Arterial Pressure, *SD* Standard Deviation, *SpO*_*2*_ peripheral capillary Hb-Saturation with oxygen measured by pulse oximetry, *SVV* Stroke Volume Variation

^†^recorded hourly in each patient’s medical record; ^‡^values obtained hourly by reading values from the ventilator

## Discussion

This study investigated how the personalised PEEP, determined via the EIT-guided titration, affected the EIT-derived parameters of ventilation homogeneity. We compared this personalised PEEP with the standard PEEP set near 5 cmH_2_O, marking the start of protective ventilation in most of our patients [6]. The outcome we studied, quantified by the respective EIT-based parameters, can answer some questions regarding the setting of PEEP titration and its necessity during controlled ventilation, taking into account the patient’s lung condition and other relevant parameters.

We observed that the PEEP titration procedure itself led to statistically significant changes in some parameters of lung ventilation homogeneity (specifically, GIi, RVDI and 4QIi). Contrary to that, the subsequent adjustments of PEEP to the recommended values found by PEEP titration did not result in statistically significant changes in any EIT-derived parameter in our cohort of 55 patients, where the change in PEEP adjustment did not exceed three cmH_2_O. This indicates that such small adjustments in PEEP may not significantly affect ventilation homogeneity despite the PEEP titration procedure indicating that these PEEP changes are needed to achieve a better balance between lung overdistension and collapse. The effect of PEEP titration on measured EIT parameters could be complex. It is expected to offer improvements in ventilation, provided PEEP levels derived from it and set on the ventilator affect the parameters of lung ventilation homogeneity in a statistically significant way. However, this was not observed in our cohort, as demonstrated by the analysed EIT-based parameters. Thus, the role of PEEP titration, if linked with its step where the initial PEEP is adjusted to the optimal value identified by it, seems questionable based on our findings and the range of PEEPs values studied (differing by no more than three cmH_2_O from the PEEP levels before the titration).

The procedure used in our study to find the optimal PEEP was decremental PEEP titration, with PEEP stepped down from 9 to 2 cmH_2_O, searching for pressure at which there was a minimal amount of collapse and overdistension as observed by EIT imaging [42]. Each step of PEEP titration lasted 50 s, which was considered sufficient regarding stabilising the EIT-derived parameters (over 50 s, the EIT-derived parameters of ventilation homogeneity reliably levelled off based on our observations). In [52], it is discussed that a duration of at least ten breaths or 30 s at each PEEP step is sufficient for a reasonably accurate estimate of compliance change since the occurrence of airway closure is reasonably fast. Still, if a PEEP is titrated with respect to gas exchange parameters with longer levelling, three minutes might be insufficient [53, 54].

It should be noted here that PEEP values at the crossing point of a minimal amount of collapse and overdistension, obtained during the PEEP titration procedure examining patients receiving myorelaxant agents (in our case, the Atracurium administered minutes before the PEEP titration), may not be optimal for patients with spontaneous breathing, exhibiting higher trans-alveolar distending pressures, different effect of PEEP on tidal volume distribution [55, 56] across various ventilatory modes [57] and higher alveolar recruitment in dorsal regions. As there were some spontaneous breaths detected by visual inspection of our data, occurring sporadically during readout times before the PEEP titration and after the PEEP titration in 7 patients, we adjusted the readout times by a few minutes to obtain readout periods without spontaneous breaths. An interesting effect, which we think could be attributed to the use of myorelaxant Atracurium, was a statistically significant drop of MAP by 3 mmHg over the course of PEEP titration and subsequent change of PEEP (see Table 4 and Fig. S8).

A PEEP of 5 cmH_2_O is the recommended value with which controlled ventilation should be conducted in acute brain tissue damage patients to minimise cerebral damage, respecting the brain-protective ventilation approach [6, 11, 18]. It states that avoiding high PEEPs could prevent cerebral hypoperfusion and reduce the risk of brain injury, a statement which applies rather to PEEPs higher than 5 cmH_2_O [23]. According to the available literature, the impact of PEEP on intracranial pressure depends on the gap between baseline intracranial pressure and baseline CVP [58]. This means that if the PEEP is already below the level of intracranial pressure, further reduction may not confer additional advantages [1]. Considering that the effect of PEEP on intracranial pressure is small, as long as the PaCO_2_ is well controlled [59], decreasing the PEEP below 5 cmH_2_O could be questionable [60], rendering the benefits of PEEP levels lower than 5 cmH_2_O still theoretical. Values as high as 10 cm could be used if intracranial pressure is not increased or the lungs exhibit low compliance values [6]. A recent meta-analysis focusing on low PEEPs in acute brain injury patients, including 2448 patients with PEEPs lower than 5 cmH_2_O, and 2957 patients with PEEP higher than that, did not prove any significant differences in analysed parameters, including patient outcomes, between these two groups [61]. Another study [62] found no significant differences in postoperative pulmonary complications when comparing PEEP levels below 5 cmH_2_O with those set at 12 cmH_2_O. Similarly, no variations in mortality rates were observed between higher (9.6 ± 3.4 cmH_2_O) and lower (1.9 ± 2.6 cmH_2_O) PEEPs, as reported in [63]. A recent investigation involving patients with acute brain injury showed no significant changes in cerebral oxygenation and related parameters between PEEP levels of 5 cmH_2_O and 15 cmH_2_O [64].

The importance of ventilation homogeneity could be questioned when SpO_2_ and pCO_2_ are well controlled to prevent secondary brain damage, assuming the FiO_2_ is within an acceptable range. We presume that ventilation homogeneity may not be a critical problem, as long as the lungs are healthy. However, implementing protective lung ventilation strategies, comprising low tidal volume, optimised levels of PEEP and recruitment manoeuvres, will likely mitigate the risk of secondary pulmonary damage attributable to significant inhomogeneities [2, 5, 16, 17]. Considering that higher PEEPs could increase intracranial pressure and interfere more adversely with patient status in acute brain injury [21, 65], reducing PEEP to values below 5 cmH_2_O could be regarded as a viable ventilation strategy for these patients. However, ensuring that such reductions in PEEP do not significantly compromise their ventilation homogeneity is crucial and deserves further investigation and empirical validation.

While numerous trials and experimental studies utilise the EIT technology primarily to investigate parameters associated with states and outcomes of acute respiratory distress syndrome [57, 66], fewer explore bedside changes in ventilation homogeneity related to the PEEP titration and PEEP adjustments in mechanically ventilated patients with healthy lungs [67–69]. To our knowledge, this study is the first to investigate the effect of PEEP titration and subsequent PEEP adjustment on ventilation homogeneity measured by the EIT in adult acute coma patients in neurocritical care with healthy lungs under mechanical ventilation.

We can either view the PEEP titration manoeuvre and the subsequent PEEP adjustment as a single composite procedure or consider them separately. When viewed as a single composite intervention, only the 4QIi (four quadrants ventilation inhomogeneity index) showed statistically significant changes. Considering them separately, the PEEP titration alone resulted in more pronounced and statistically significant changes in lung ventilation homogeneity. This could be interpreted as an effect of the initial phase of titration elevating the PEEP to 9 cmH_2_O, a level likely inducing moderate recruitment [38], as well as an effect of possible derecruitment caused by the last phase of titration decreasing the PEEP to 2 cmH_2_O, both very likely altering the proportion of collapses and overdistension, or other lung parameters influencing the measured parameters of ventilation homogeneity. However, the subsequent adjustment of PEEP did not cause any statistically significant changes in these parameters in our cohort, likely due to the relatively small changes in PEEP brought by the PEEP adjustment as already discussed (standard deviation of ∆PEEP was 1.96 cmH_2_O in our cohort; see Table 2 for details).

Another possible explanation for the negligible, statistically non-significant changes observed in measured parameters following the adjustment of PEEP levels could be a loss or vanishing of possible recruiting effect of PEEP titration [70–72]. This derecruitment, occurring over time intervals ranging from minutes to tens of minutes, could lead to the lungs being in different conditions many minutes after the PEEP titration, thus depreciating the “optimality” of PEEP values identified by the PEEP titration. Since in our cohort of 47 patients undergoing the PEEP titration and subsequent PEEP adjustment, the median time between the end of PEEP titration and the PEEP adjustment was 18 min (see Table 2), we tried to analyse this possible derecruitment. We processed subgroups of patients whose PEEP was adjusted sooner than 20 min (20 patients, see Fig. S9) and sooner than 10 min (9 patients, not shown) after the end of PEEP titration. In both cases, non-significant changes in EIT-derived parameters of ventilation homogeneity were found due to the sequence of both the PEEP titration and the subsequent change of PEEP. Thus, a derecruitment following the PEEP titration was not likely the reason behind our findings, although analogous effects with similar dynamics cannot be excluded since they can begin minutes after recruiting manoeuvres and exhibit a rather complex time dynamic [38, 46, 71].

Our results suggest that the PEEP titration procedure and setting of the optimal PEEP derived from it may not significantly improve ventilation homogeneity in healthy lungs when changes in PEEP are less than three cmH_2_O, as observed in our study.

Our study has strengths and limitations. Among its strengths is the processing of high temporal resolution EIT data (update rate 20 ms). This allowed us to analyse the EIT-based indexes of ventilation homogeneity calculated for each breath, evaluated at specific readout times and averaged over suitable time intervals. Among its limitations is the small change in PEEP level brought by PEEP adjustment, reducing the scope of examined PEEPs resulting from the PEEP titration (the maximum change in PEEP at PEEP adjustment was three cmH_2_O, with a mean of |∆PEEP|= 1.7 cmH_2_O). Also, we have not systematically assessed in each patient which PEEP values probed during the PEEP titration procedure were most optimal measured by EIT-based parameters of ventilation homogeneity or whether they would result in improved ventilation homogeneity. Moreover, we refrained from making any qualitative interpretations regarding the changes in measured indexes of ventilation inhomogeneity and associating them with better or worse ventilation distribution in mechanically ventilated patients. We just used these measures to quantify how much the optimal PEEP setting obtained from the PEEP titration procedure affects these EIT measures. Another limitation was the presence of sporadic spontaneous breaths during the controlled ventilation detected in seven patients during the study protocol. Although the readout intervals were successfully shifted by a few minutes to exclude these spontaneous breaths, any undetected spontaneous breathing drive could have slightly altered the measured parameters. Furthermore, we only refer to PIP values instead of plateau pressures, which may not be an optimal surrogate for driving or transpulmonary pressure, especially when considering patient breathing effort. Additionally, static compliance was recorded by nurses only once per hour, and we lacked sufficient time resolution in recorded pressure curves to derive it from plateau pressures or P–V diagrams breath-wise. Given the size and heterogeneity of our population, we believe it was sufficient to support some generalisations regarding the measured changes in ventilation homogeneity caused by the PEEP adjustments in healthy lungs, provided the PEEP changes will be similarly small. To generalise the findings related to temporal dynamics of measured parameters of ventilation homogeneity, a larger sample size or more controlled conditions would be required to strengthen some of our negative conclusions regarding the time separation between the PEEP titration and the subsequent PEEP adjustment. Another limitation of our study, especially when compared to many experimental animal studies, was the challenge we faced in strictly adhering to the examination protocol to minimise the variance of certain explanatory variables, such as tidal volume, driving pressure, and time separating the PEEP titration and the subsequent change of PEEP. Autonomous ventilation settings could have altered the first two parameters, and despite the variance in these parameters not being substantial, as demonstrated by our data, we attempted to address that through post-hoc analyses on different subgroups of patients having these parameters within specific ranges.

## Conclusions

1. Our data indicate that titrating the PEEP using the EIT method to minimize overdistension and collapse and subsequently applying this titrated PEEP to mechanically ventilated patients with healthy lungs does not offer a clear advantage over using a standard PEEP of 5 cmH_2_O, particularly if the PEEP change derived from the titration is not bigger than three cmH_2_O.
2. Regarding neurointensive care patients undergoing mechanical ventilation, our findings indicate that if EIT-based PEEP titration identifies an optimal PEEP of less than 5 cmH_2_O, it is unlikely to significantly affect ventilation homogeneity provided the change in PEEP is not bigger than 3 cmH_2_O and the initial PEEP set to 5 cmH_2_O. Such low PEEP levels may offer potential benefits in managing neurocritical care patients and mitigate risks of secondary brain damage.

The abovementioned conclusions were drawn from analysing a cohort of 55 acute neurocritical care patients undergoing mechanical ventilation, all with healthy lungs. These patients were managed using pressure control regimes ASV or DouPAP, with lung ventilation homogeneity evaluated by EIT-derived parameters.

### Supplementary Information

Supplementary material 1.

## Data Availability

The data that support the findings of this study are available on request from the corresponding author (VS).

## Abbreviations

ASV: Adaptive support ventilation
bpm: Breaths per minute
C_dyn_: Dynamic lung mechanical compliance
C_el_: Electrical compliance
CI: Cardiac index
C_stat_: Static lung mechanical compliance
CVP: Central venous pressure
DuoPAP: Duo positive airway pressure
EIT: Electrical impedance tomography
EtCO_2_: End-tidal partial pressure of CO_2_
FiO_2_: A fraction of inspired oxygen
GIi: Global inhomogeneity index
NICU: Neuro-intensive care unit
MAP: Mean arterial pressure
MV: Mechanical ventilation
PaCO_2_: Partial pressure of CO_2_ in arterial blood
PEEP: Positive end-expiratory pressure
PIP: Peak inspiratory pressure
RR: Respiratory rate
RVDI: Regional ventilation delay inhomogeneity
SpO_2_: Peripheral capillary Hb-Saturation with oxygen measured by pulse oximetry
SVV: Stroke volume variation
T_aPA+5min_: Readout time 5 min after the PEEP adjustment to optimal values
T_bPA_: Readout time 2 min before the PEEP adjustment
T_bPT_: Readout time 5 min before the beginning of PEEP titration
VT: Tidal volume
4QIi: Four Quadrants ventilation Inhomogeneity index
