# Supplementary material for: Standard versus individualised positive end-expiratory pressure (PEEP) compared by electrical impedance tomography in neurocritical care: a pilot prospective single centre study
Source: Intensive Care Med Exp. 2024 Aug 5;12:67. doi: 10.1186/s40635-024-00654-3 (PMC11300775; doi:10.1186/s40635-024-00654-3)
Supplement: Supplementary file 1 — Supplementary material 1. [file 40635_2024_654_MOESM1_ESM.docx]

Fig. S1 GIi of the right and left lungs, electrical compliance of entire lungs.


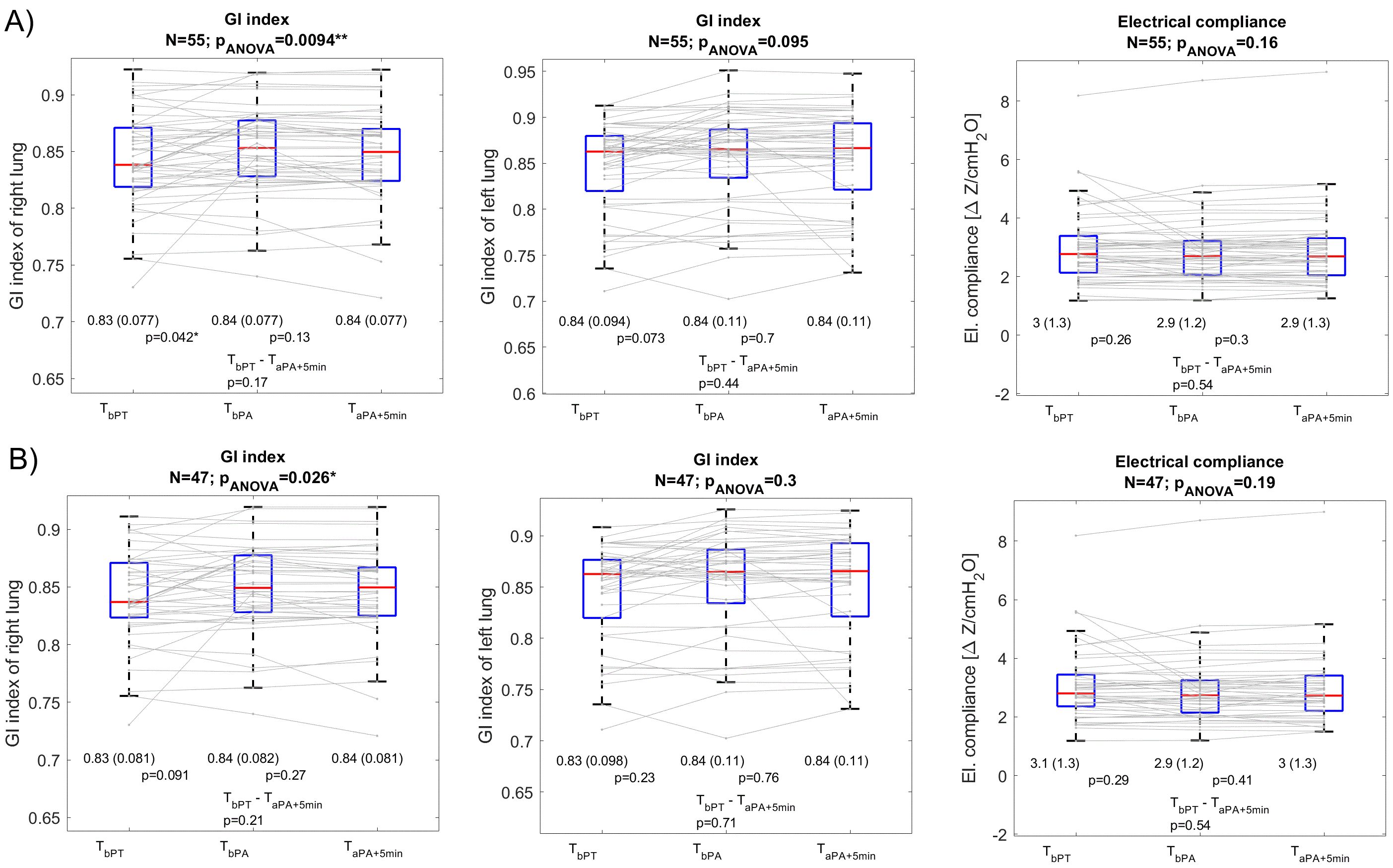


Boxplots show how much the GIi (Global Inhomogeneity index) of the right and left lung, as well as the electrical compliance of the entire lungs, changed between measurements taken 5 minutes before the PEEP titration (T_bPT_), 2 minutes before the PEEP adjustment (T_bPA_), and 5 minutes after the PEEP adjustment (T_aPA+5min_). Panel A shows the parameters for all 55 enrolled patients. Panel B shows the parameters for a subgroup of 47 patients whose PEEP value changed after the PEEP titration due to the subsequent PEEP adjustment.

Fig. S2 Eight patients whose PEEP stayed unchanged after the PEEP titration


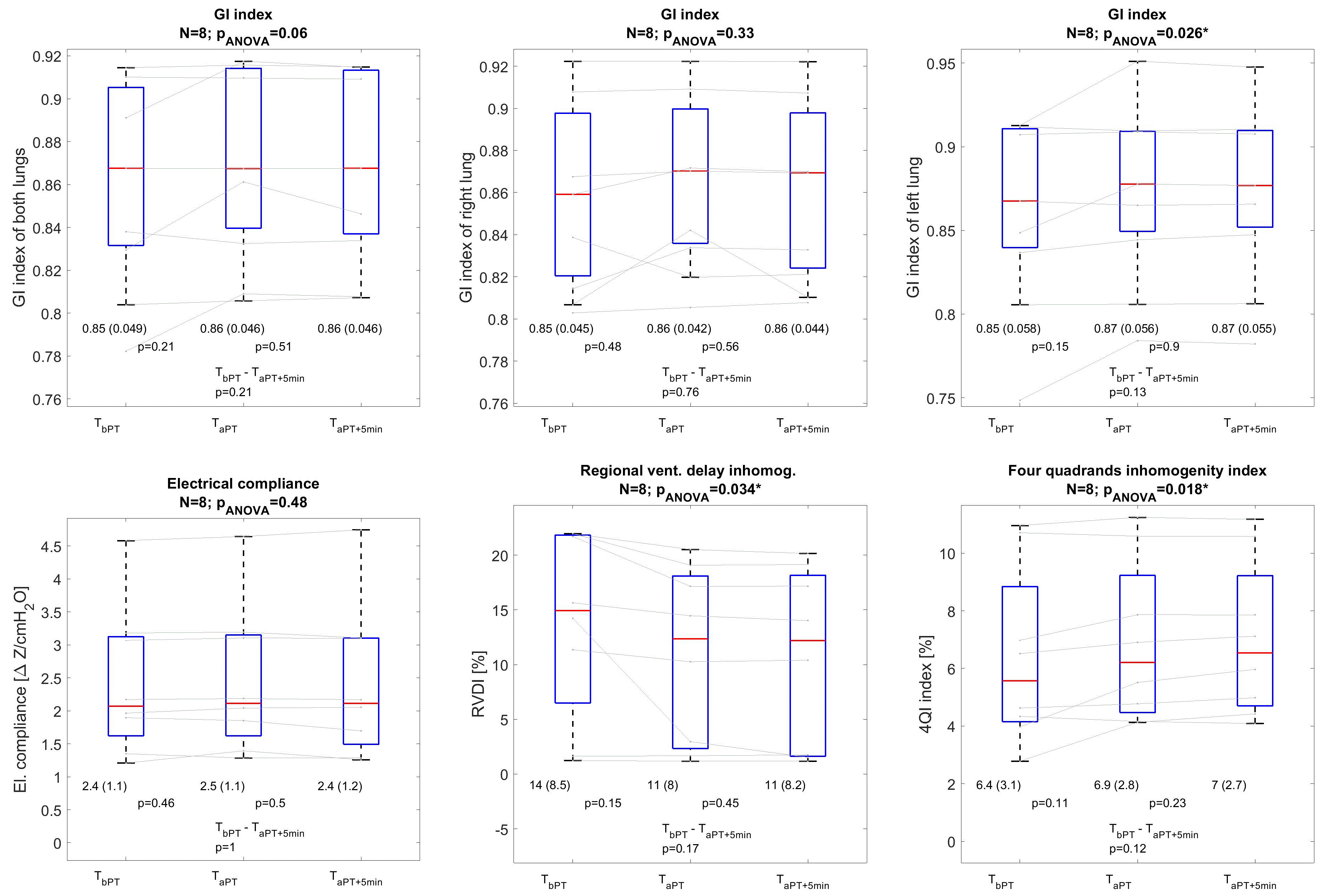


The PEEP was not adjusted in these patients after the PEEP titration procedure because it indicated that the current PEEP was already optimal. Boxplots show how much the EIT-derived parameters changed between measurements taken 5 minutes before the PEEP titration (T_bPT_), 1 minute after PEEP titration (T_aPT_), and 5 minutes after the PEEP titration (T_aPT+5min_). The

GI - Global Inhomogeneity, RVDI - Regional Ventilation Delay Inhomogeneity; 4QI - Four Quadrants ventilation Inhomogeneity

Fig. S3 Subgroup of 16 patients with DUOPAP ventilation regime


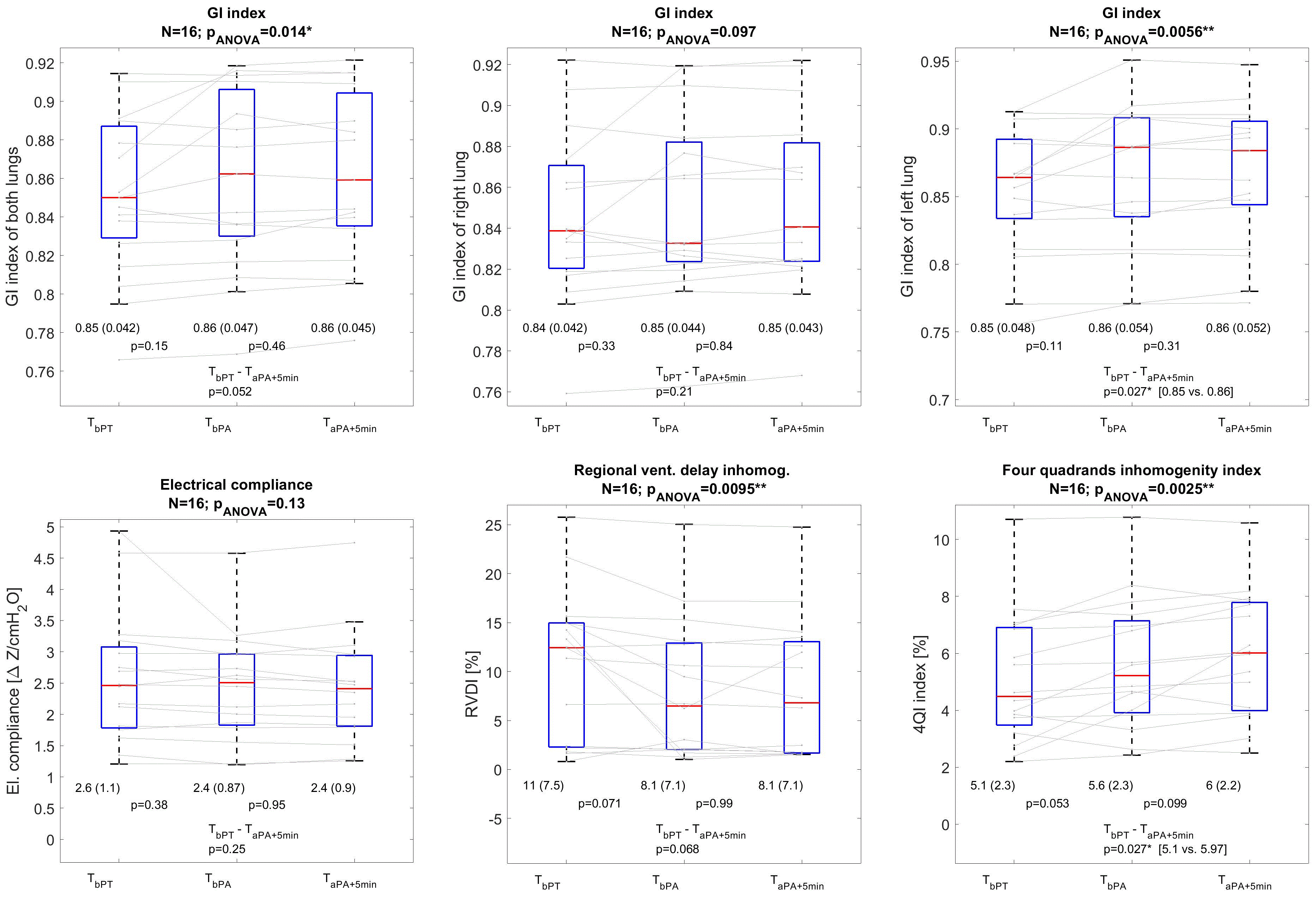


Boxplots show how much the EIT-derived parameters changed between measurements taken 5 minutes before the PEEP titration (T_bPT_), 2 minutes before the PEEP adjustment (T_bPA_), and 5 minutes after the PEEP adjustment (T_aPA+5min_).

GI - Global Inhomogeneity, RVDI - Regional Ventilation Delay Inhomogeneity; 4QI - Four Quadrants ventilation Inhomogeneity

Fig. S4 Subgroup of 39 patients with ASV ventilation regime


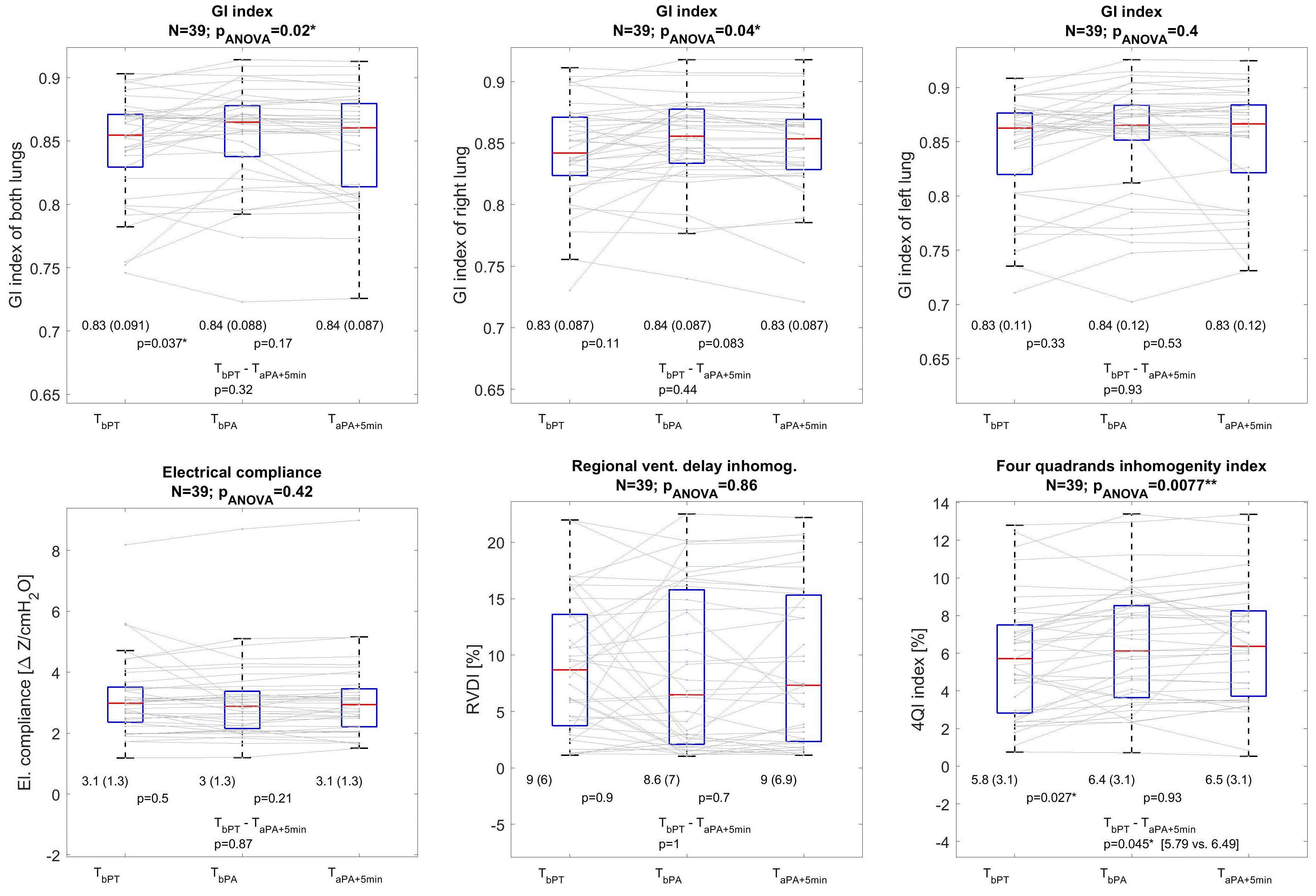


Boxplots show how much the EIT-derived parameters changed between measurements taken 5 minutes before the PEEP titration (T_bPT_), 2 minutes before the PEEP adjustment (T_bPA_), and 5 minutes after the PEEP adjustment (T_aPA+5min_).

GI - Global Inhomogeneity, RVDI - Regional Ventilation Delay Inhomogeneity; 4QI - Four Quadrants ventilation Inhomogeneity

Fig. S5 Subgroup of 28 patients whose tidal volume changed by less than 20 mL between the T_bPT_ and T_aPA+5min_


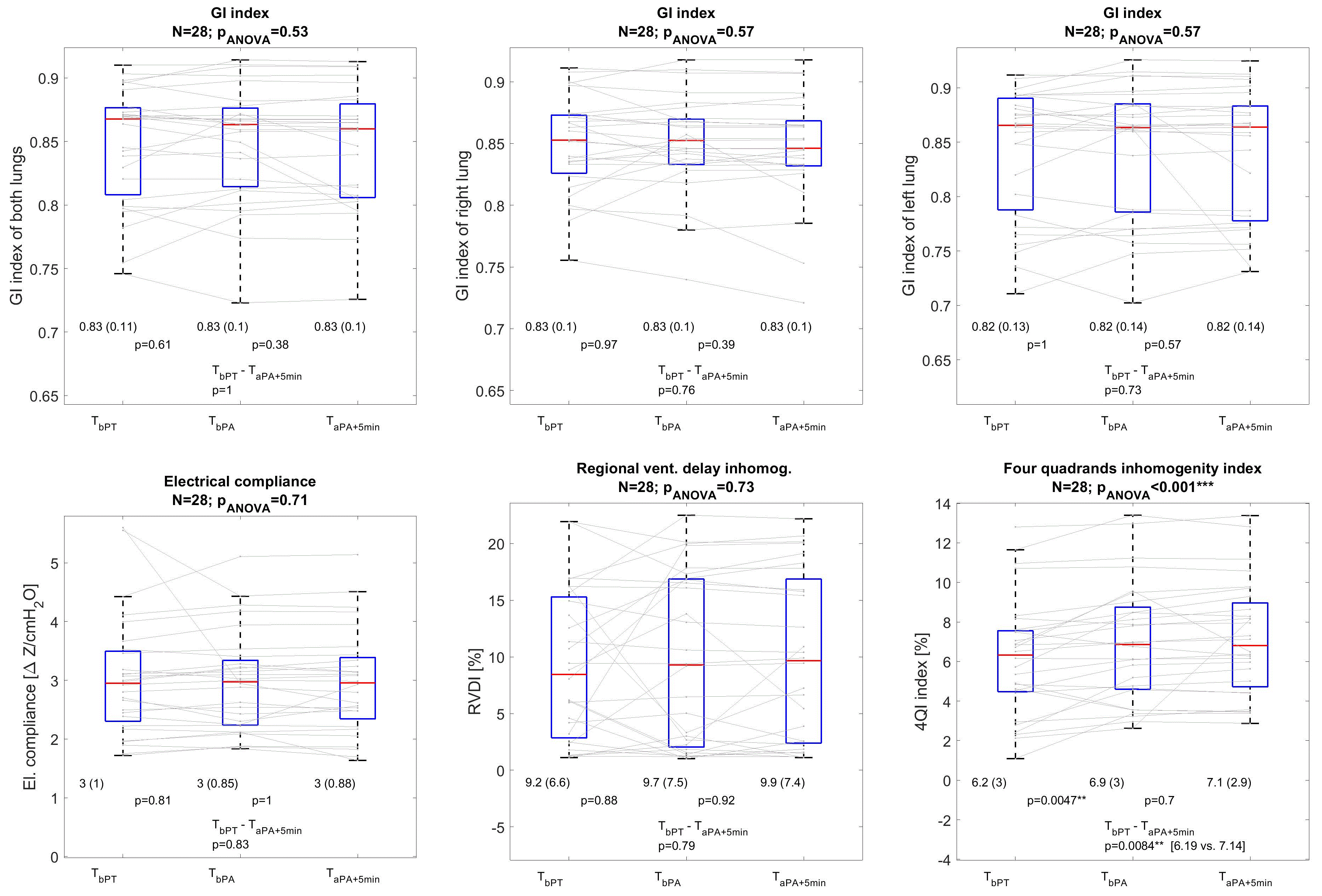


Boxplots show how much the EIT-derived parameters changed between measurements taken 5 minutes before the PEEP titration (T_bPT_), 2 minutes before the PEEP adjustment (T_bPA_), and 5 minutes after the PEEP adjustment (T_aPA+5min_).
GI - Global Inhomogeneity, RVDI - Regional Ventilation Delay Inhomogeneity; 4QI - Four Quadrants ventilation Inhomogeneity

Fig. S6 Subgroup of 27 patients whose tidal volume changed by more than 20 mL between the T_bPT_ and T_aPA+5min_


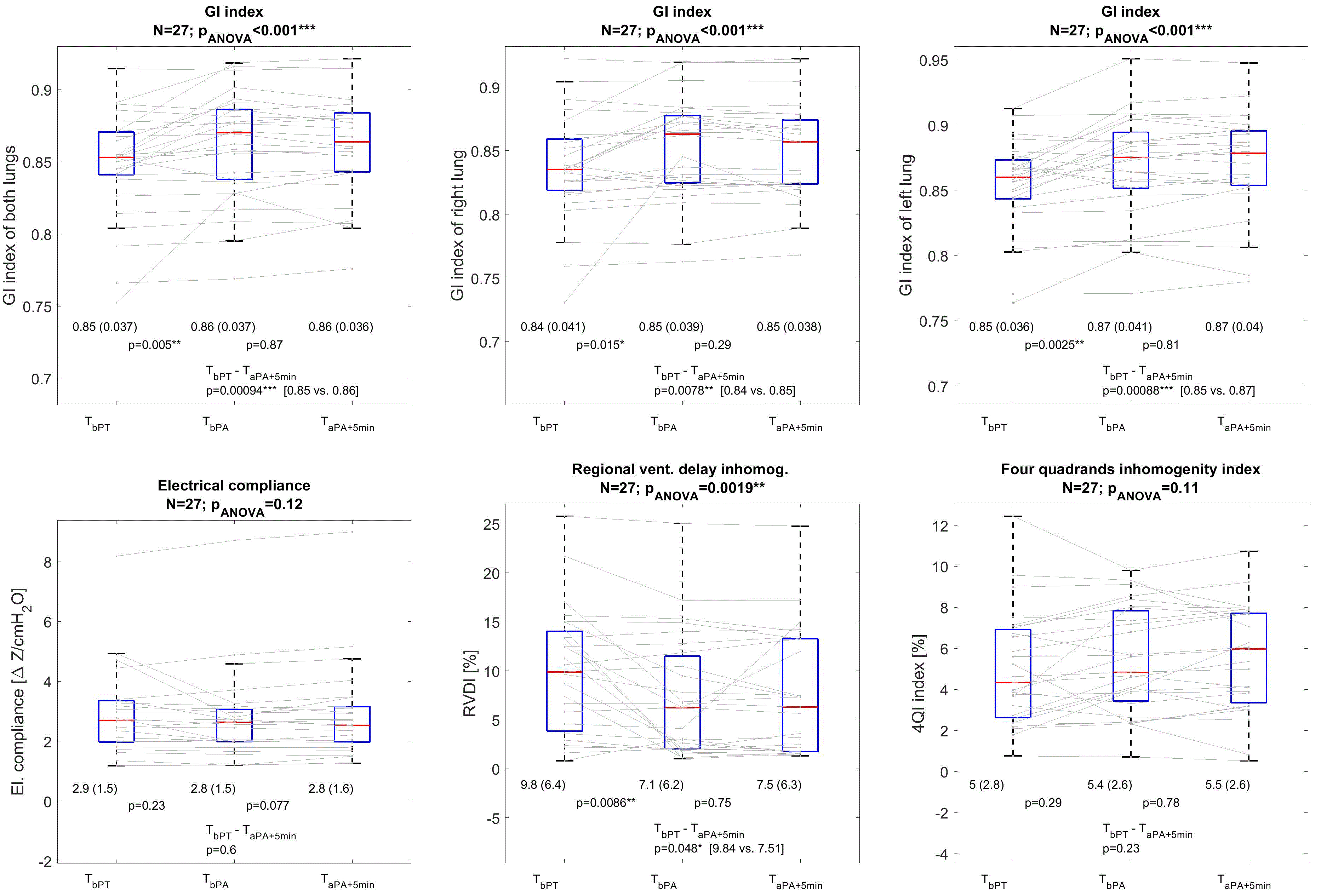


Boxplots show how much the EIT-derived parameters changed between measurements taken 5 minutes before the PEEP titration (T_bPT_), 2 minutes before the PEEP adjustment (T_bPA_), and 5 minutes after the PEEP adjustment (T_aPA+5min_).
GI - Global Inhomogeneity, RVDI - Regional Ventilation Delay Inhomogeneity; 4QI - Four Quadrants ventilation Inhomogeneity

Fig. S7 Subgroup of 14 patients whose PEEP changed by two and more cmH_2_O between the T_bPT_ and T_aPA+5min_ (absolute value of ΔPEEP is ≥ 2 cmH_2_O)


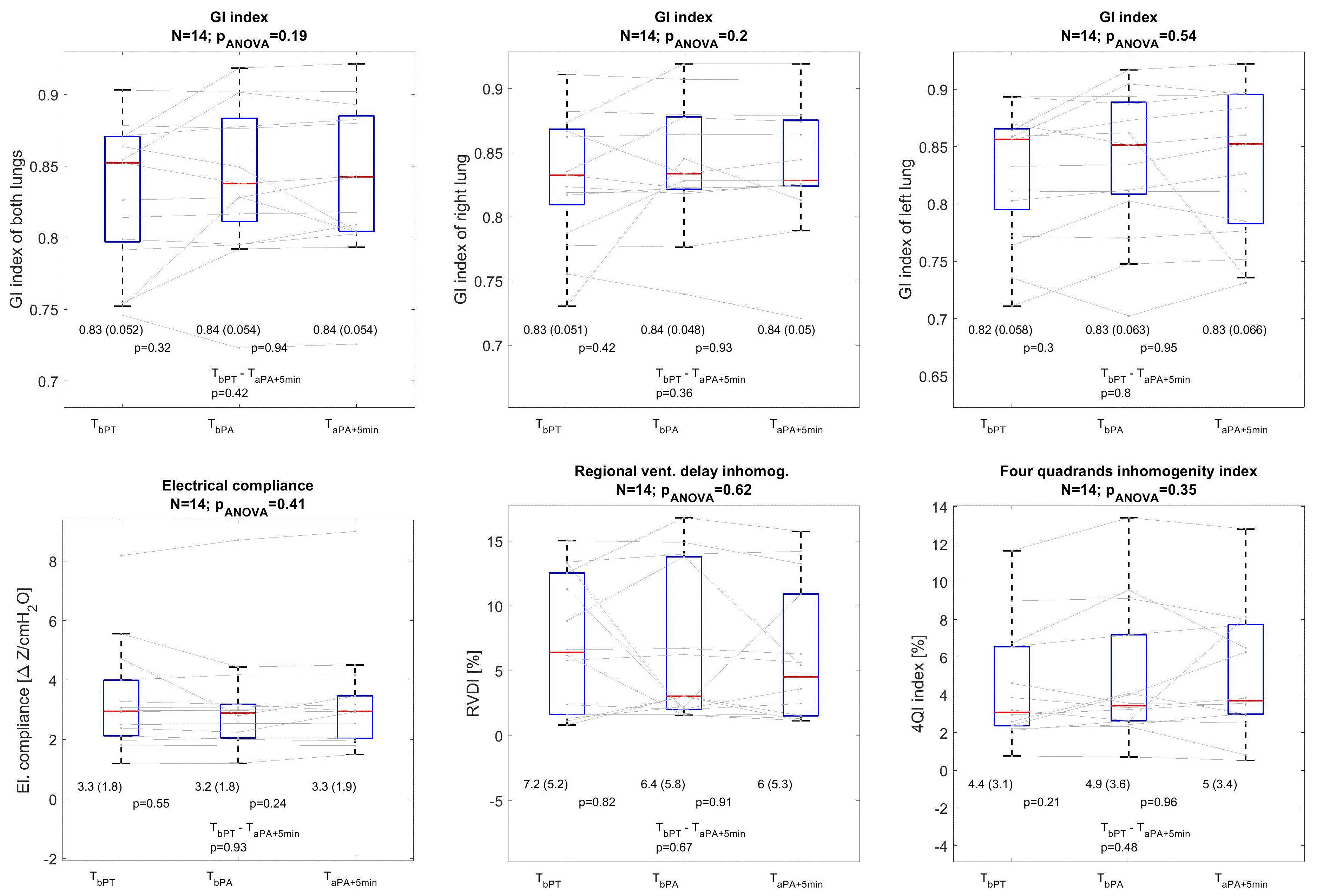


Boxplots show how much the EIT-derived parameters changed between measurements taken 5 minutes before the PEEP titration (T_bPT_), 2 minutes before the PEEP adjustment (T_bPA_), and 5 minutes after the PEEP adjustment (T_aPA+5min_).
GI - Global Inhomogeneity, RVDI - Regional Ventilation Delay Inhomogeneity; 4QI - Four Quadrants ventilation Inhomogeneity

Fig. S8 The change in parameters recorded manually by nurses
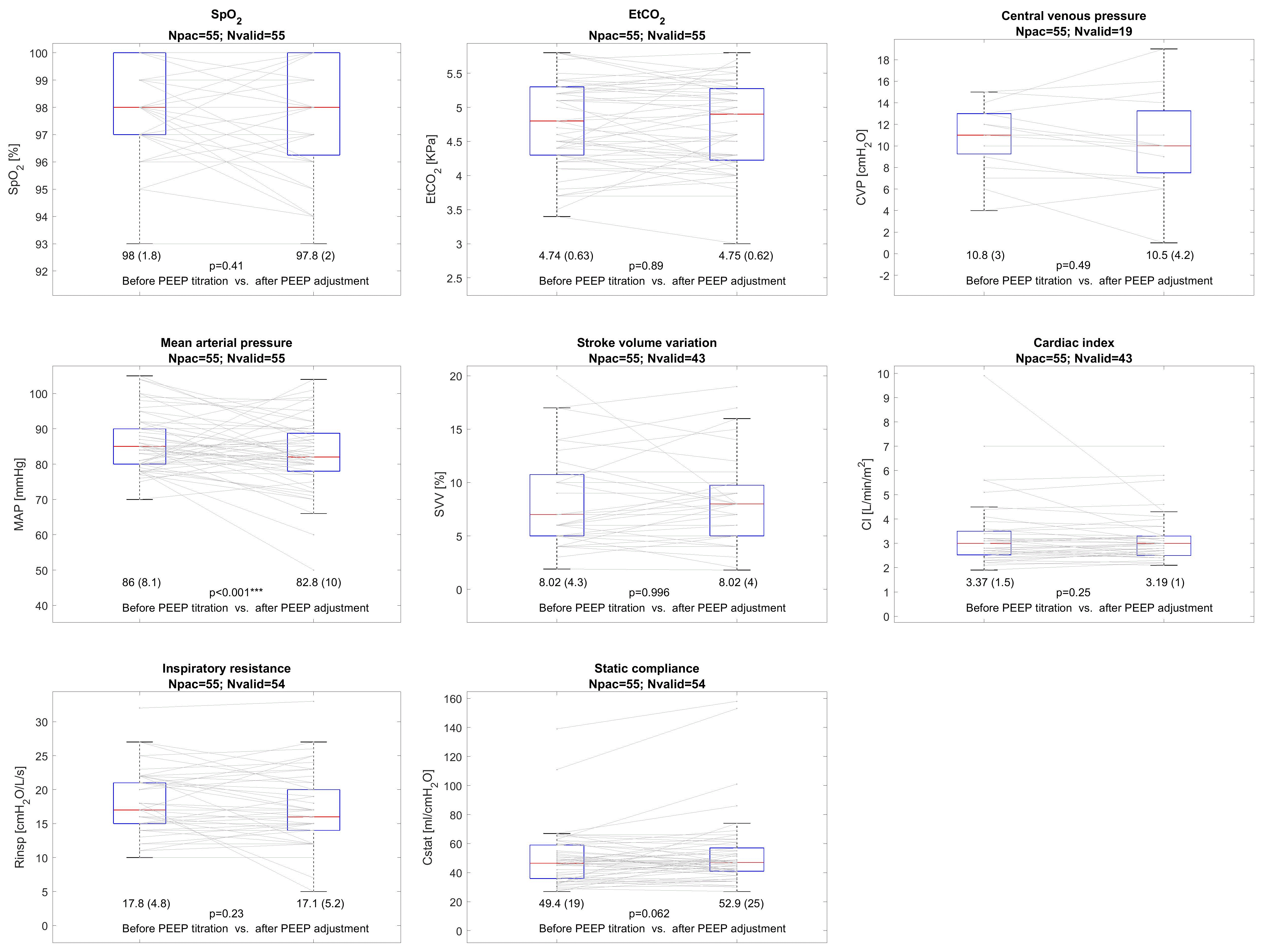


Boxplots show how the hourly recorded parameters changed due to both effects of PEEP titration and adjustment of PEEP. The readout times could have occurred at any point within one hour before or after the PEEP titration. Thus, these manually recorded parameters were obtained with much coarser temporal resolution than EIT-derived parameters. N_valid_ represents the number of patients with an existing value of a given parameter before the PEEP titration and after the PEEP adjustment. Paired t-test was used to calculate *p*-values.

Fig. S9 Subgroup of 20 patients with PEEP change occurring sooner than 20 min after the end of PEEP titration


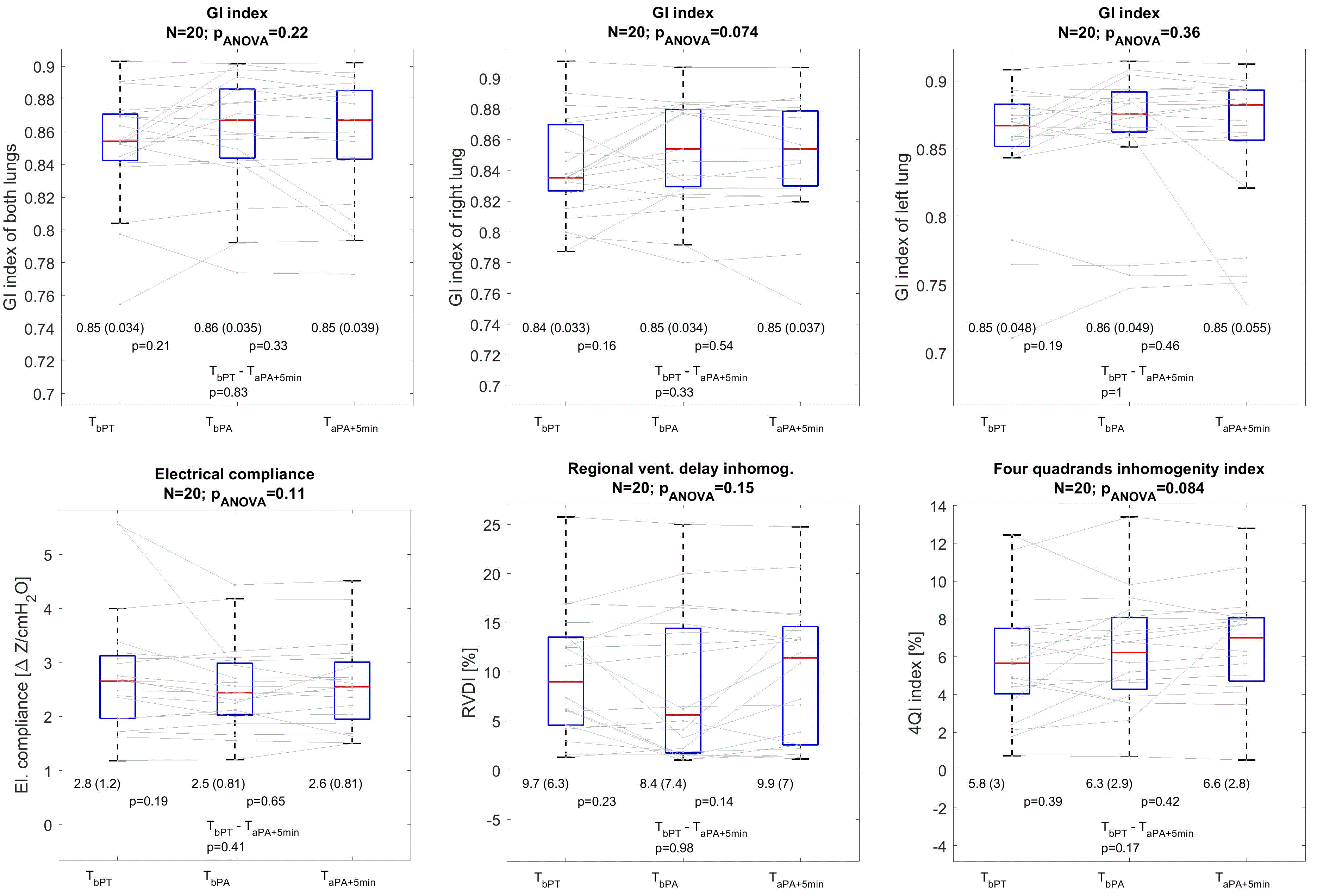


Boxplots show parameters of 20 patients whose time separation between the end of PEEP titration and the change of PEEP was smaller than 20 min. Measurements were taken 5 min before the PEEP titration (T_bPT_), 2 min before the PEEP adjustment (T_bPA_), and 5 min after the PEEP adjustment (T_aPA+5min_).

GI - Global Inhomogeneity, RVDI - Regional Ventilation Delay Inhomogeneity; 4QI - Four Quadrants ventilation Inhomogeneity

Table S1 The effect of PEEP titration and subsequent adjustment of PEEP in various subgroups of patients

| Parameter | *p*-values of Tukey’s multiple comparison tests: | | | | | |
| --- | --- | --- | --- | --- | --- | --- |
| GIi both lungs  GIi right lung  GIi right lung  RVDI both lungs  4QIi  C_dyn_  PEEP  PIP-PEEP  VT | Comparison between groups of patients ventilated with ASV (39 pts) and with DuoPAP regimes (16pts) | | | | | |
|  | T_bPT_ vs. T_aPA+5min_^†^ | | T_bPT_ vs. T_bPA_^‡^ | | T_bPA_ vs. T_aPA+5min_^§^ | |
|  | ASV | DuoPAP | ASV | DuoPAP | ASV | DuoPAP |
|  | 0.32  0.44  0.93  0.99  0.045*  0.99  0.80  0.25  0.061 | 0.052  0.21  0.027*  0.068  0.027*  0.99  0.0072**  0.0041**  0.0013** | 0.037*  0.11  0.33  0.90  0.027*  0.82  0.16  0.11  0.69 | 0.15  0.33  0.11  0.071  0.053  0.90  0.08  0.17  0.045* | 0.17  0.08  0.53  0.10  0.70  0.93  0.92  0.78  0.92 | 0.46  0.84  0.31  0.99  0.099  0.93  0.69  0.74  0.62 |
|  | Change in selected parameters of VT and PEEP between respective readouts T_bPT_, T_bPA_ and T_aPA+5min_ | | | | | |
| Mean of ΔVT  SD of ΔVT  SD of ΔPEEP | ↑ 7 mL  48 mL  1.6 cmH_2_O | ↑ 54 mL  49 mL  1.4 cmH_2_O | ↑ 6 mL  49 mL  0.4 cmH_2_O | ↑ 36 mL  55 mL  0.5 cmH_2_O | ↑ 1 mL  19 mL  1.9 cmH_2_O | ↑ 18 mL  73 mL  Two cmH_2_O |
|  | | | | | | |
| GIi both lungs  GIi right lung  GIi right lung  RVDI both lungs  4QIi  C_dyn_  PEEP  PIP-PEEP | Comparison of subgroups of patients with \|ΔVT\|, \|ΔPEEP\|, or both parameters within a specified range between T_bPA_ and T_aPA+5min_ | | | | | |
|  | T_bPA_ vs. T_aPA+5min_ | | | | | |
|  | \|ΔVT\| < 20 mL  28 pts | \|ΔVT\| > 20 mL  27 pts | \|∆PEEP\|< 2 cmH_2_O  41 pts | \|∆PEEP\|≥ 2 cmH_2_O  14 pts | \|ΔVT\| < 20 mL and  \|∆PEEP\|≥ 2 cmH_2_O  5 pts | \|ΔVT\| > 20 mL and  \|∆PEEP\|≥ 2 cmH_2_O  9 pts |
|  | 0.38  0.39  0.57  0.92  0.70  0.40  0.98  0.88 | 0.87  0.29  0.81  0.75  0.78  0.057  0.99  0.99 | 0.23  0.10  0.50  0.32  0.17  0.8  0.99  0.96 | 0.94  0.93  0.95  0.91  0.96  0.23  0.98  0.74 | 0.79  0.99  0.82  0.97  0.95  0.98  0.99  0.38 | 0.92  0.91  0.49  0.71  0.99  0.22  0.93  0.87 |
|  | Change in selected parameters of VT and PEEP between T_bPA_ and T_aPA+5min_ readouts | | | | | |
| Mean of ΔVT  SD of ΔVT  SD of ΔPEEP | ↑ 3 mL  22 mL  1.9 cmH_2_O | ↑ 15 mL  56 mL  2.1 cmH_2_O | ↓ 2 mL  33 mL  1.6 cmH_2_O | ↑ 15 mL  51 mL  2.9 cmH_2_O | ↑ 10 mL  15 mL  3.7 cmH_2_O | ↑ 40 mL  61 mL  2.5 cmH_2_O |

**p*-value <0.05, ***p*-value <0.01, ****p*-value <0.001

^†^Comparison between parameters 5 minutes before PEEP titration and 5 minutes after PEEP adjustment

^‡^Comparison between parameters 5 minutes before PEEP titration and 2 minutes before PEEP adjustment

^§^Comparison between parameters 2 minutes before PEEP adjustment and 5 minutes after PEEP adjustment

ΔPEEP, ΔVT – the change in respective parameters between two given readouts, |ΔVT|, |∆PEEP| - absolute values of the change between two given readouts
ASV - Adaptive Support Ventilation, DuoPAP - Duo Positive Airway Pressure, GIi - Global Inhomogeneity index, PEEP - Positive End-Expiratory Pressure; PIP - Peak Inspiratory Pressure, RVDI - Regional Ventilation Delay Inhomogeneity, SD – Standard Deviation, T_bPT_ – readout time 5 min before the PEEP titration, T_bPA_– readout time 2 min before the PEEP adjustment, T_aPA+5min_ – readout time 5 min after the PEEP adjustment, VT – Tidal Volume, 4QIi - 4 quadrants ventilation inhomogeneity index
